# Open preperitoneal versus anterior approach for recurrent inguinal hernia: a randomized study

**DOI:** 10.1186/1471-2482-12-22

**Published:** 2012-10-30

**Authors:** Aly Saber, Goda M Ellabban, Mohammad A Gad, Karam Elsayem

**Affiliations:** 1 Department of general surgery, Port-Fouad General Hospital, Port-Fouad, Port-Said, Egypt; 2 Department of surgery, Faculty of medicine, Suez Canal University, Ismailia, Egypt

## Abstract

**Background:**

Inguinal herniorrhaphy remains one of the most common general surgical operations, with approximately 15% performed for recurrence. The repair of the resulting recurrent hernia is a daunting task because of already weakened tissues and obscured and distorted anatomy. The aim of this study is to compare the posterior preperitoneal versus anterior tension-free approach for repair of unilateral recurrent inguinal hernia regarding complications and early recurrence.

**Methods:**

120 Patients in this study were divided randomly into 2 main groups; Group A patients were subjected to posterior preperitoneal approach and those of group B were subjected to conventional anterior tension-free repair. The primary end point was recurrence and the secondary end points were time off from work, postoperative pain, scrotal swelling and wound infections.

**Results:**

The mean hospital stay was 1.2 days and 4.7, the mean time to return work was 8.2 and 11.2 days and the mean time off from work was 9.4 and 15.9 days in group A and B respectively. The maximum follow-up period was 48 months and the minimum was 14 months with a mean value as 37.11 ± 5.14 months. Only 2 recurrences (3.3%) in group A and 4 cases (6.25%) in group B were seen. The final pain score per patient and the overall complication rate were higher in group B.

**Conclusions:**

The open preperitoneal repair offers the advantages of low recurrence rate and allows covering all potential defects with one piece of mesh and is far superior to the anterior approach.

**Trial Registration:**

ACTRN12611000337976

## Background

Inguinal herniorrhaphy remains one of the most common general surgical operations, with approximately 8-17% performed for recurrence as reported in nationwide large scale Danish study [1,2]. There is little available evidence on the optimal management of recurrent inguinal hernia, particularly if the original procedure involved the use of mesh [3]. The choice of an optimal strategy and surgical technique is probably more important in the treatment of recurrent hernias than in other areas of hernia surgery [4]. The repair of the resulting recurrent hernia is a daunting task because of already weakened tissues and obscured and distorted anatomy. The failure rate of these repairs using an open anterior approach may reach as high as 36% [5]. The evolution of the posterior preperitoneal approach for recurrent inguinal hernia repair made it the procedure of choice for the management of all recurrent groin hernias [6].

Some surgeons recommend laparoscopic repair of recurrent inguinal hernias whereas others prefer an open repair but preferences are not based on large-scale data and their repair usually depends on local expertise, economical considerations and patient preference [2]. Laparoscopic repair is preferred in patients with a previous open repair, while patients with recurrence after laparoscopic repair should undergo open mesh repair [1]. In contrast, laparoscopic operation of a recurrence after a primary laparoscopic repair provided no statistical advantage in terms of lower re-reoperation rate compared with all open techniques [1,2]

The aim of this study was to compare the open posterior preperitoneal versus anterior tension-free approach for repair of unilateral recurrent inguinal hernia regarding complications and early recurrence.

## Methods

### Patients

This study represented parallel prospective randomized clinical trial where patients were divided randomly into two main groups; A and B. Group A patients were subjected to open posterior preperitoneal approach and those of group B were subjected to transinguinal anterior tension-free repair. All of our patients were gentlemen with total number was 120 patients; 60 for each group, their ages ranged between 42–65 years. The study started from January 2007 to December 2009 and included all patients having unilateral recurrent inguinal scrotal and irreducible hernias. Patients with primary inguinal hernia, patients with marked obesity (BMI > 35) and ASA grade 3 and beyond were excluded.

### Sample size

In general, the overall complications of transinguinal anterior tension-free repair for recurrent hernia reported in previous studies is about 40% (7) and those of open posterior preperitoneal approach is about 17% (8). Calculation of the sample size included the number of participants to be recruited for the study using the mathematical equation. The authors used these two equations to calculate the minimum number required to reliably answer the research question. Using the first equation (9), the number, m = ~ 50 patients for each group, as given by:

(1) $${\frac{2\times \left[\text{Z}\left(1-\text{ά}/2\right)\text{+Z}\left(1-\text{β}\right)\right]}{{\text{Δ}}^{2}}}^{2}=\;50\;\text{patients}$$

where z (1- a/2) and z (1- **β**) represent percentage points of the normal distribution for statistical significance level (**ά)** at 0.05 value is 1.96 and power (1-**β)** with accepted 95% positive rate is 1.6449, where β, the false-negative rate. **Δ** represents the standardized difference (i.e. the treatment difference divided by its standard deviation.

(2) $$\text{Standardized difference}\;\Delta =\frac{P^{1}-P^{2}}{\sqrt{\Sigma x\left(1–\Sigma \right)}}$$

(3) $$\text{Where}\;\Sigma =\frac{P^{1}-P^{2}}{2}$$

**P**^**1**^ represents the overall complications of transinguinal anterior tension-free repair reported in previous studies = 40% (7).

**P**^**2**^ represents the overall complications of open posterior preperitoneal approach reported in previous studies = 17% (8).

The sample size was calculated according to the second equation (10).

(4) $$\text{m=}\frac{\text{K}\left({\text{p}}_{1}{\text{q}}_{1}+{\text{p}}_{2}{\text{q}}_{2}\right)}{{\text{d}}^{2}}\text{=60 patients}$$

Where:

(5) $$\text{q1=}\left(1-\text{p}1\right),\text{q2=}\left(1-\text{p}2\right)\;\text{and d=}\left(\text{p1-p}2\right)$$

**K =** constant, which depends on: alpha and beta levels, where alpha =0.05 and beta =0.1. Then K =8.6.

### Randomization

Randomization was performed prior to study commencement as follows: Opaque envelopes were numbered sequentially from 1 to 120. A computer-generated table of random numbers was used for group assignment; if the last digit of the random number was from 0 to 4, assignment was to Group A (posterior preperitoneal approach), and if the last digit was from 5 to 9, assignment was to Group B (anterior tension-free repair). The assignments were then placed into the opaque envelopes and the envelopes sealed. As eligible participants were entered into the trial, these envelopes were opened in sequential order to give each patient his or her random group assignment. The envelopes were opened by the operating surgeon after patient consent and just prior to the surgery.

### Surgical teams & study sites

Operations were performed in Port-Fouad general hospital, Port-Fouad, Port-Said, Egypt and in the university hospital, department of surgery, Faculty of medicine, Suez Canal University, Egypt.

### Operative techniques

1- The open preperitoneal approach to the inguinal region was performed under general or regional anesthesia, as originally described by Nyhus [6]. Through a lower abdominal transverse incision, the anterior rectus sheath was incised and the rectus muscle reflected medially. The preperitoneal space was cleaved with blunt dissection, exposing the myopectineal orifice. The cord was explored and the hernias were reduced. A 15×15 cm polypropylene mesh with a slit was inserted in the preperitoneal space and fixed with nonabsorbable sutures to pubic tubercle and Cooper's ligament. The mesh was passed behind the cord and manipulated to lay flat against the posterior inguinal floor overlapping the entire myopectineal orifice.

2- The anterior tension-free repair, as defined by Lichtenstein et al. [1] was performed using 6 × 11 cm polypropylene mesh. Large pore-sized (1.6 mm), monofilament heavy-weight polypropylene meshes were used (Prolene®; Ethicon, Egypt). Really our patients were oriented to the type of repair and the other observers were unaware to operative techniques of the study groups.

### End points

The primary end point of the study was recurrence of the hernia, defined as a clinically detectable characteristic swelling in the groin and diagnosed by the two authors. The secondary end points were time off from work, defined as the number of days between the day of surgery and the first day a patient returned to work, postoperative pain, scrotal swelling and wound infections. Regarding the postoperative pain, we considered the Visual Analog Scale pain score, prosthesis awareness and return to normal physical activity. Chronic pain was defined as pain lasting more than 3 months and was studied in relation to age, body mass index and operative procedure [7].

Here we adopted a simplified scoring system for method of pain assessment. This system is a 3-scale system; with maximum score as 7 points and minimum as 2 points.

1- Analog Scale pain score: (1–10) Mild (1–4) =1 point, moderate (5–7) = 2 points, severe (8–10) = 3 points.

2- Prosthesis awareness: yes = 1 point, no = 0 point.

3- Physical activity: pain only on exertion = 1 pain limits some daily activity = 2, disabling pain = 3.

### Ethical consideration

Written consents were obtained from all patients before the study. The steps of both operative interferences were explained to all patients. The local ethics committee had approved all operative procedures. Ethical approval for this study was granted by the ethical review committee under supervision of the general director of Port- Fouad general hospital, Port-Fouad, Port-Said, Egypt.

### Statistical analysis

The statistical tests were run on a compatible personal computer using the Statistical Package for Social Scientists (SPSS) for windows 15. Chi-square distribution was used for studying the frequencies of recurrence, pain, hospital stay and postoperative complications. The values were expressed as means ± standard errors of deviation. The mean values of the groups were compared by one-way analysis of variance (ANOVA) and paired comparisons of the groups were done using the paired student *t* test. P < 0.05 was considered significant.

## Results

There was no statistical difference between the two groups as regard age and body mass index (Table 1). Age ranged between 42 – 65 years with a mean age as 53.5 years. Follow-up assessment was at the 1st week after discharge then at 1st month and through regular visit of 6 months duration or by a telephone call thereafter. Follow up included patients’ complaint, if any, clinical examination and ultrasonography if needed. The maximum follow-up period was 48 months and the minimum was 14 months with a mean value as 37.11 ± 5.14 months. A complete follow-up was obtained in 56/60 (93.3%) of patients in group A and 54/60 (90%) of patients in group B.

**Table 1:** Showing subdivision of both groups regarding age and body mass index

**Group**	**Age < 50**	**Age >50**	**BMI < 25**	**BMI < 30**	**BMI > 30**
	**42-----49**	**50----65**	**22---24.9**	**25---29.9**	**30---34.9**
**A**	N = 38	N = 22	N = 16	N = 24	N = 20
**B**	N = 36	N = 24	N = 18	N = 23	N = 19

The mean operative time in group A was 71.6 min ± 25.47 (40–120). In group B, the mean value was 94.7 min ± 28.5 (60–150) with insignificant distribution [p = 0.7].

The morphology of hernia recurrence during surgery in patients of group A was as follow:

1- Pubic tubercle recurrence, 12/60 (20%).

2- Internal ring recurrence, 24/60 (40%).

3- Total posterior wall recurrence, 24/60 (40%).

The morphology of hernia recurrence during surgery in patients of group B was as follow:

4- Pubic tubercle recurrence, 24/60 (40%).

5- Internal ring recurrence, 20/60 (33.33%).

6- Total posterior wall recurrence, 16/60 (26.67%).

The mean hospital stay was 1.2 (1–3) days in group A and 3.7 (2–6) in group B. In the other hand, the mean time to return work was 8.2 (7–10) days in group A while in group B was 11.2 (7–15). So, the mean time off from work in group A was 9.4 days and in group B was 14.9 [P < 0.05].

Chronic postoperative pain was observed in 8 patients in group A (13.33%) and in 18 patients in group B (30%). Table 2 shows the detailed descriptions of pain in both groups as well as the final pain score per patient [P < 0.003]. The authors found that 6/8 patients in group A were in the age group < 50 years and the other two were > 50 year while regarding the body mass index, all 8 patients were < 30. In group B, 12/18 patients were in the age group < 50 and BMI < 25 kg/m2 and the remaining 6 were in the age group > 50 and BMI < 30 kg/m2. All patients who were aware of the presence of prosthesis and pain on exertion belonged to the smaller age group < 50 and the less BMI < 25 kg/m2.

**Table 2:** Showing the detailed descriptions of pain in both groups as well as the final pain score per patient during the follow-up period

**Group**	**VAS**		**Prosthesis awareness:**		**Physical activity:**		**Total score**	**Score per patient**
A N = 8 (13.3%)	Mild	7	yes	6	pain on exertion	2	17	2.1
	mod	1	no		pain limits daily activity			
	severe	-			disabling pain			
B N = 18 (30%).	mild	6	yes	10	pain on exertion	6	54	3
	mod	8	no		pain limits daily activity	2		
	severe	4			disabling pain			

Regarding the early postoperative complications, 6 patients (10%) in group A developed mild scrotal swelling due to tissue oedema not for haematoma formation and wound seroma formation while in group B, the figure was higher as 12 patients (20%) experienced mild- to moderate scrotal swelling and seroma. Wound infection was seen in 3 patients (5%) in both groups necessitating only dressing in the outpatient clinic under cover of systemic antibiotics with no need to remove the mesh but those patients developed re-recurrence thereafter. Testicular atrophy was not seen and only 2 recurrences (3.3%) in group A all over the period of follow up. Five cases of testicular atrophy (8.3%) and 4 cases of hernia recurrence (6.25%) were seen in group B {P ≥ 0.05}. Moreover, the overall postoperative complication rates were 18.3% and 40% in group A and group B respectively with significant distribution {P < 0.01} (Table 3).

**Table 3:** Showing the detailed descriptions of results in both groups with corresponding p-values

**Points**	**Group A**	**Group B**	**P value**
**Operative time**	40-120 min 71.6 ± 25.47	60-150 min 94.7 ± 28.5	NS
**Hospital stay**	1-3 days	2-6 days	P < 0.05
**Time off from work**	9.4	14.9	
**Pain score**	2.1	3	P < 0.003
**Early complications**	6	12	P < 0.01
Wound infection	3	3	
**Late complications:** a- testicular atrophy	-	5	
b- hernia recurrence	2	4	

NS = non significant.

## Discussion

In the present study, the authors succeeded to establish follow up of their patients to 56/60 (93.3%) in group A and 54/60 (90%) in group B for a mean period of 37.11 ± 5.14 months. These figures are comparable to those in studies of the same interest; 40, 31.3 and 32 months respectively [8-10] as these periods of follow up seem sufficient to detect late complications as testicular atrophy and hernia recurrence [7].

The morphology of recurrence in anterior approach during surgery showed that pubic tubercle recurrence was the most common form (40%) and the posterior wall recurrence represented (26.67%) cases of recurrence. Anatomo-clinical classification of recurrences can help the surgeon in individuating the choice of operation [4]. In preperitoneal repair group, the morphology of recurrence was a bit different from that in the other group as the internal ring and total posterior wall recurrence were equally seen. In other studies of same interest, The majority of recurrences were medial or suprapubic [11] and medial, lateral or combined [12].

The most effective method to repair an inguinal hernia in any given patient is not clearly defined. The repair of recurrent inguinal hernia after mesh repair is usually a difficult operation due to the disadvantage of reoperating through dense fibrotic scar tissue around the mesh with the risk of testicular damage and a large number of local haematoma [8,9,13].

The open posterior preperitoneal mesh repair was popularized by Nhyus [6] as a good alternative for recurrent inguinal hernias. The main advantages of the preperitoneal approach are mesh placement in the preperitoneal space where the hernia is produced and avoiding the disadvantage of reoperating through scar tissue [8,14,15]. From the molecular point of view, the approach to the inguinal canal through the preperitoneal space appears less invasive than the transinguinal anterior approach where TNF-alpha levels are highest in the open anterior group [16].

In the present study, we found that the open posterior preperitoneal approach really reduced the time of hospital stay and sick leaves and accordingly the time off from work compared with the anterior approach and the difference was statistically significant. Many studies of same interest reported less hospital stay and rapid return to physical activity [8,17,18].

Chronic postoperative pain is strongly related to two main patient-related factors; age and body mass index [19-21] or three surgery- related factors [22] such as surgery for recurrence with anterior approach [8,14,15], operations performed in specialist hernia centers [23] and finally the experience of the surgeon [24]. The open posterior preperitoneal approach in the present study significantly reduced the final chronic pain score per patient in comparison with the anterior approach. An interesting study found that surgery for recurrent hernia significantly increased the risk of chronic pain 4-fold more than primary repair [19]. Also, surgery for a recurrent hernia showed a significant higher incidence of moderate or severe chronic pain 12 months after operation compared with primary herniorrhaphy [19,25].

There are many studies compared patient age with the occurrence of chronic pain and found that the risk of chronic pain decreased with increasing age [19,20,25,26]. Our data regarding this point came in concordance with these reports. The BMI is another studied factor for chronic pain occurrence where many investigators found good correlation between less BMI values and chronic pain [27-29] and our data in the present study supported these finding.

The postoperative complications of hernia repair were estimated regarding the rate and traced regarding the type in similar previous studies as early and delayed forms [6,9,13,14,30]. Early complication, defined as that occurring within one month of surgery, are wound seroma, sepsis, scrotal oedema and haematoma formation while the long-term complications, assessed at three months are testicular atrophy and recurrence [31,32].

Wound haematoma and superficial wound infections are the most common problems in previous series and serious complications, such as major hemorrhage, pubic osteitis and testicular atrophy, occur in fewer than 1 percent of patients undergoing herniorrhaphy [32]. The overall postoperative complication rates were 18%-38% [32] and may reach as high as 49.7% in some series [31] and in our study, the overall complication rate was 19.7% in group A and 34.36% in group B due to more tissue dissection and manipulation. Minor complications such as seroma formation, wound sepsis and scrotal haematoma were seen in both groups in the present study but with more incidence in group B. This observation met with data reported by other investigators [7,30]. Long term complications such as testicular atrophy and recurrence were traced by many researchers who reported 0% incidence for testicular atrophy and 0% or very low incidence (1.5%) for recurrence in their studies [7,9,14,28] while others found 4.38% hernia recurrence after posterior preperitoneal repair [15] 10% in the open anterior approach [33,34] while other data ranged between 2% -14% [32]. Accordingly, we found 2 cases of recurrence in group A one of them was a gentleman with heavy work and body mass index 32 kg/m2 and suffering from wound sepsis while the 4 patients in group B were from the morphological and anatomo-clinical classification of total posterior wall recurrence. The re-recurrence of both groups as the primary end point of our study was statistically studied as individual item. The recurrence rate of both groups was statistically insignificant despite being higher in those of group B than in group A. Nyhus in his original report found 3% recurrence after preperitoneal approach [6] while other researchers on studying the approach for inguinal hernia recurrence found 4,4% recurrence in both preperitoneal and anterior approaches [4].

Testicular atrophy and necrosis as a result of ischemic orchitis is a well-established complication after anterior inguinal hernia repair with 1% occurrence following primary herniorrhaphy and 5% in recurrent cases [35-37] but in open preperitoneal repair, the procedure is safe as it effectively eliminates testicular complications [7,30]. Testicular ischemia and necrosis is thought to be due to acute thrombosis of the pampiniform venous plexus rather than arterial injury, as there is collateral arterial flow to the testis from the inferior epigastric, vesical, prostatic and scrotal arteries [36]. Testicular atrophy is thought to be more common after open procedures particularly recurrent inguinal hernias due to greater manipulation of the spermatic cord beyond the pubic tubercle and during dissection of the distal hernia sac [37]. According to these finding, we found that no testicular atrophy was seen in patients of group A but seen in 2 patients of group B (4.25%) due to operating within the fibrotic field with tissue reaction around the mesh.

Farook and colleagues [14] in similar randomized clinical trial found that hospital stay and return to normal activity were similar for both groups. Regarding recurrence, they found no recurrence in either group after a mean follow-up of 2 years. But in the present study and in others of same interest [8,17,18], the time of hospital stay and the time off from work were less in posterior preperitoneal repair groups. In the authors' own opinion, this discrepancy may be owed to increased overall complication rates in these studies.

## Conclusions

The open preperitoneal hernia repair offers many advantages. It is inexpensive, has a low recurrence rate, and allows the surgeon to cover all potential defects with one piece of mesh. Postoperative recovery is short and postoperative pain is minimal. This approach gives results far superior to those of the commonly used anterior approach (See Additional file 1).

## Competing interests

The authors declare that they have no competing interests.

## Authors’ contributions

AS conceptualized the proposal of the article. All authors performed the surgical operation as the surgical team. AS & GME: got acquisition of data through web. MAG & KE conducted the statistical analyses AS carried out the manuscript drafting. MAG & GME revised the manuscript and put the final approval. All authors read and approved the final manuscript.

## Pre-publication history

The pre-publication history for this paper can be accessed here:

http://www.biomedcentral.com/1471-2482/12/22/prepub

## Supplementary Material

Additional file 1 CONSORT 2010 Flow Diagram. Click here for file
