# Supplementary material for: Open preperitoneal versus anterior approach for recurrent inguinal hernia: a randomized study
Source: BMC Surg. 2012 Oct 30;12:22. doi: 10.1186/1471-2482-12-22 (PMC3514268; doi:10.1186/1471-2482-12-22)
Supplement: Additional file 1 — CONSORT 2010 Flow Diagram. [file 1471-2482-12-22-S1.doc]

**CONSORT 2010 Flow Diagram**

**Allocation**

**Analysis**

**Follow-Up**

**Enrollment**

Assessed for eligibility (n=120 )

Excluded (n= )

♦  Not meeting inclusion criteria (n= )

♦  Declined to participate (n= )

♦  Other reasons (n= )

Analysed (n= 56 )
♦ Excluded from analysis (give reasons) (n=0 )

Lost to follow-up (give reasons) (n=4 ) Reasons for this loss: Cultural aspect and moving to another residence

Discontinued intervention (give reasons) (n= )

Allocated to intervention (n=60 ) Group A patients were subjected to open posterior preperitoneal approach ♦ Received allocated intervention (n= )

♦ Did not receive allocated intervention (give reasons) (n= )

Lost to follow-up (give reasons) (n= 6 ) Reasons for this loss: Cultural aspect and moving to another residence

Discontinued intervention (give reasons) (n= )

Allocated to intervention (n=60 ) group B were subjected to transinguinal anterior tension-free repair.

♦ Received allocated intervention (n= )

♦ Did not receive allocated intervention (give reasons) (n= )

Analysed (n=54 )
♦ Excluded from analysis (give reasons) (n=0 )

Randomized (n=120 ) patients were divided randomly into two main groups; A and B. patients were divided randomly into two main groups; A and B. patients were divided randomly into two main groups; A and B.
